# Psycho-social experience of oocyte recipient women: a qualitative study

**DOI:** 10.1186/s12905-021-01562-4

**Published:** 2021-12-09

**Authors:** Shohre Ghelich-Khani, Ashraf Kazemi, Malek Fereidooni-Moghadam, Mousa Alavi

**Affiliations:** 1 School of Nursing and Midwifery, Isfahan University of Medical Sciences, Isfahan, Iran; 2 Nursing and Midwifery Care Research Center, School of Nursing and Midwifery, Isfahan University of Medical Sciences, Hezarjerib AV., Isfahan, Iran

**Keywords:** Oocyte donation, Psychological reaction, Stigma, Qualitative study

## Abstract

**Background:**

Although associated with many successes, oocyte donation can lead to numerous psychological challenges in recipient women. The identification of these challenges during the treatment process is crucial to improve recipient mental health. Thus, the aim of this study was to gain an understanding of the experiences of oocyte recipient women.

**Methods:**

This research was conducted using a qualitative approach and inductive content analysis method. The data collection tool was in-depth interviews. Twenty women with the experience of receiving donated oocyte were selected and entered the study using purposive sampling method and considering the maximum variation.

**Results:**

Three main categories of psychological challenges were extracted from patient interviews, specifically, distressing psychologic symptoms, social stigmatization, and negative coping mechanisms. The category of distressing psychologic symptoms was shaped based on the subcategories of self-esteem destruction, anxiety and stress, depression and spiritual discouragement. The category of social stigmatization included the subcategories of concern about disclosure, judgment of others, and conflict with religious teachings. And the category of negative coping mechanisms was formed based on the subcategories of aggression and denial.

**Conclusion:**

The results indicated that the process of treatment with donated oocyte is followed by the experiences of distressing psychologic symptoms, social stigmatization, and negative coping mechanisms in recipient women. As such, paying attention to the socio-cultural factors which affect this process seems necessary to maintain the mental health of these women.

**Plain English summary:**

Although associated with many successes, oocyte donation can lead to numerous psychological challenges in recipient women. The aim of this study was to gain an understanding of the experiences of oocyte recipient women. This research was conducted using a qualitative approach and inductive content analysis method. The data collection tool was in-depth interviews. Twenty women with the experience of receiving donated oocyte were selected and entered the study using purposive sampling method and considering the maximum variation. Three main categories of psychological challenges were extracted from patient interviews, specifically, distressing psychologic symptoms, social stigmatization, and negative coping mechanisms. The category of distressing psychologic symptoms was shaped based on the subcategories of self-esteem destruction, anxiety and stress, depression and spiritual discouragement. The category of social stigmatization included the subcategories of concern about disclosure, judgment of others, and conflict with religious teachings. And the category of negative coping mechanisms was formed based on the subcategories of aggression and denial. The results indicated that the process of treatment with donated oocyte is followed by experience of distressing psychologic symptoms, social stigmatization, and negative coping mechanisms in recipient women. As such, paying attention to the socio-cultural factors which affect this process seems necessary to maintain the mental health of these women.

## Background

With the prevalence of 21% [2], infertility is a major global issue which has been increasingly growing in recent years all over the world [1]. Associated with an unexpected stress, infertility can cause marital problems for infertile couples [3]. Inner desire for having children on the one hand, and its unattainability on the other, pave the way for many social problems including increased rate of divorce [6], polygamy in some societies [7], social isolation and stigma [8], domestic violence [5], and the feelings of guilt and worthlessness as well [9].

All of these problems can increase psychological symptoms such as anxiety and depression in couples [4] and negatively affect their quality of life [10]. Therefore, infertile couples may try to overcome this problem by using assisted reproductive techniques. Lack of response to medical treatment and non-existence of potentially fertile oocytes make these couples to resort to donated oocytes [11]. Although the use of this treatment method has brought about significant successes, the lack of woman's genome sharing in reproduction can pose significant challenges to couples, especially women. Accordingly, some infertile couples, who have received oocytes, refer to this event as a "tragedy" in their life [12].

The reluctance of the recipient couples, especially women, to disclose the resulting offspring's genetic origin [13], concerns about the physical and mental health of the donor [14], decreased self-concept [15], concerns about the outcome of the treatment [16], ethical and religious issues [17], and economic and social challenges [18] are among the most known problems of using this technique. Furthermore, the significance of fertility as the most prominent gender-related role of women in traditional societies leads to new dimensions of social challenges for women in these societies [19].

Higher rate of depression in women than men in such societies [4], attributing infertility to women [20] and the taboo of female infertility [21] are among the evidence showing that women face more major problems in these countries. As such, the experience of using oocyte donation can affect these women more than others. Moreover, men have already played their gender role in the reproduction of the resulting offspring via gamete sharing [11].

However, the non-participation of women in the fetus's genetic makeup together with socio-cultural challenges they face can impair their mental health [22]. Therefore, pregnancy of these women is completely different from that of other women who have become pregnant naturally or by using assisted reproductive techniques through autologous oocytes. This considerably unique experience has been overlooked by researchers. Therefore, the aim of the present study was to gain an understanding of the experiences of oocyte recipient women.

## Methods

This qualitative study was conducted using inductive content analysis approach [23].

### Participants

This qualitative study was conducted on 20 Iranian recipients of donated oocytes who had been referred to public and private infertility centers of Isfahan, Iran. These women were in one of the stages of the assisted reproductive technique, namely, the treatment process, waiting for the pregnancy test results, and either successful or unsuccessful results of treatment. The inclusion criteria were the ability to communicate properly, having ability to express their feelings and experiences, and having no severe and persistent mental illness according to the recognition of a psychiatrist and the documentations of the medical records.

### Data collection

The participants were selected using purposive sampling method from August 2019 to February 2020 in Isfahan, Iran. The data collection technique in this study was interview. The interviews were conducted following a semi-structured and in-depth script. According to the ethics committee of Isfahan University of Medical Sciences guidelines, after introducing herself and the objectives of the research, the researcher invited the oocyte recipients to participate in the study. Sampling was performed by presenting the university's letter of introduction to the officials of infertility services and obtaining a license from them. The participants were identified in two ways. First, a number of participants, who were undergoing assisted reproductive techniques through using donated oocyte, were identified based on their medical records. Second, those who had been treated for the past two years were identified and selected by evaluating their archived records.

After identifying the participants and evaluating their inclusion criteria, the eligible ones were invited through a telephone call to participate in the study, and the necessary explanations were provided to them. After obtaining informed oral consent, the time and place of the interview were determined considering the participants' opinions.

Sampling and interviews were conducted by a member of the research team who was a PhD student in psychiatric Nursing, under the supervision of a reproductive health specialist. Based on the participants' preferences, most of the interviews were conducted in a private room in ART service centers by using a tape recorder. Before each interview, informed oral consent was obtained again for recording the voices of the participants. For the participants who did not like to record their voice, the interview was handwritten.

If women were willing to participate in the study, they were explained that their participation or non-participation will not affect their treatment process, their information will be completely confidential, and they can leave the study whenever they wish. Then, they were provided with the necessary explanations about the process of the study, and entered the study after obtaining their informed consent and the permission to record their voice.

The interviews began with guiding questions such as "explain your experience of receiving a donated oocyte;" "explain your thoughts and feelings since oocyte donation was suggested to you;" or "what issues affected your emotions?" Emotional reactions of the participants were recorded manually during the interview so that they may lead to a deeper understanding during the analysis of the interviews.

Depending on the circumstances of the participants, the duration of each interview was 60–90 min. If necessary, note taking was also performed during the interviews. Each interview was transcribed and arranged for qualitative content analysis, and the data were analyzed simultaneously. The interviews continued until categories were emerged and data saturation was reached. Data were considered saturated when no new code was extracted from the interviews and the previous codes were repeated at least 4 times [24].

### Data analysis

In parallel with data collection, the data were analyzed manually through using Graneheim and Lundman method [23]. At first, in order for the researchers to get familiar with the data, the text of each interview was read several times; then, meaning units and initial codes were extracted from the raw data. At this stage, the data were examined line by line and the codes were identified. In the next stage, the initial codes were categorized and similar codes were put in subcategories. Finally, the main categories were created based on the subcategories.

In-depth interviews with the participants, reading each interview several times and, thus, immerging in the data, together with using the opinions of expert colleagues for the confirmation or possible correction of the extracted codes and categories were used in order to increase the credibility of the data. Moreover, to assess the consensus of the researchers and participants on the codes, a number of interviews were returned to the participants after coding.

To increase of dependability, access methods, clear coding and evidence-based coding (quotations) were used. In order to ensure confirmability, the researcher used the reviews of the observers together with the supplementary opinions of four experts in the areas of psychiatric nursing, reproductive health and psychology who were skillful in the field of qualitative research. A rich descriptive presentation was provided for readers in the research report to evaluate the applicability of the data in other areas and the data transferability as well [25].

### Ethical considerations

The ethics committee of Isfahan University of Medical Sciences (IR.MUI.RESEARCH.REC.1398.312) approved the study and all methods were performed in accordance with the relevant guidelines.

## Results

A total number of 23 participants undergoing assisted reproductive treatment through using donated oocytes were invited to participate in the study, of whom 20 were interviewed. Of those who did not consent to participate in the study, one was in the phase of the donor's ovarian stimulation and the other two had not received successful assisted reproductive treatment. All three refused to participate in the study as they were concern about disclosure of their infertility treatment via using donated oocytes.

The demographic characteristics of the participants are shown in Table 1. From the analysis of the interviews with 20 Muslim participants aged between 29 and 47 years old, 98 inferential codes were extracted. After merging duplicate codes and those with the same concept, finally 90 inferential codes, 9 subcategories and 3 main categories were inferred. The three main categories included distressing psychologic symptoms, social stigmatization, and negative coping mechanisms (Table 2).

**Table 1:** Demographic characteristics of the participants

The number of the participant	Age	Education	Job	Duration of infertility	Treatment duration (years)	Successful pregnancy history
P1	41	Bachelor's degree	Employee	9	7	No
P2	39	Master's degree	Employee	9	7	No
P3	32	Middle school	Housewife	9	8	No
P4	29	Bachelor's degree	Employee	8	3	Yes
P5	30	Primary school	Housewife	10	5	Yes
P6	40	Bachelor's degree	Employee	8	8	No
P7	36	Middle school	Housewife	16	15	No
P8	47	Middle school	Housewife	2	2	Yes
P9	37	Primary school	Housewife	10	8	No
P10	41	Bachelor's degree	Employee	9	7	No
P11	47	Primary school	Employee	23	23	Yes
P12	45	Bachelor's degree	Employee	3	3	Yes
P13	44	Bachelor's degree	Retired	3	3	No
P14	44	Bachelor's degree	Employee	4	3	No
P15	38	Bachelor's degree	Employee	3	3	No
P16	40	Bachelor's degree	Employee	3	3	No
P17	35	Primary school	Employee	10	4	No
P18	35	Primary school	Housewife	9	8	No
P19	40	Middle school	Employee	16	15	No
P20	39	Master's degree	Employee	10	5	No

**Table 2:** Main and subcategories

Main categories	Subcategories
Distressing psychologic symptoms	Self-esteem destruction Anxiety and stress Depression Spiritual discouragement
Social stigmatization	Concerns about disclosure Judgment of others Contrast with religious teachings
Negative coping mechanisms	Aggression Denial

The subcategories of self-esteem destruction, anxiety and stress, depression and spiritual discouragement were merged into the distressing psychologic symptoms. The subcategories of concerns about disclosure, judgment of others and conflict with religious teachings resulted in the main category of social stigmatization. The negative coping mechanisms category was formed based on the subcategories of aggression and denial.

### Distressing psychologic symptoms

One of the main categories of the research was distressing psychologic symptoms which was inferred from the subcategories of self-esteem destruction, anxiety and stress, depression, and spiritual discouragement.

#### Self-esteem destruction

The experience of 50% of the oocyte recipients showed that upon realizing that they could not use their own oocyte for fertility and had to use a donated oocyte, they had a feeling of inferiority and defect, resulting in a loss of self-esteem which grew over time. Combined with an infertility-related sense of guilt, this feeling was intensified in these women. In this regard, participant 8 said: *"I really felt down. For my husband's sake, I had a feeling of weakness and deficiency. I was very upset that I had to implore another woman to give me her oocyte. My self-esteem was crushed and I felt my pride had left me."*

#### Anxiety and stress

Another subcategory of distressing psychologic symptoms was anxiety and stress. According to the experiences of 60% of the participants, the problem of infertility together with using a third party, donated oocyte and inflexible treatment programs, caused emotional distress such as anxiety and stress. This effect was so evident that in some cases, the treatment cycle was stopped or medication was used on the advice of a psychiatrist to reduce their anxiety. Participant 2 said in this regard: *"I felt I had no control over the newly created situation and I became more anxious upon entering a new phase of the treatment. We had problems such as lack of a specific medicine, the success or failure of the treatment, or problems associated with the cooperation of the donor; however, the doctor asked me to be not anxious. Stress and anxiety can lead to the failure of the treatment. Isn't that so? I'd like to go to a deep sleep and wake up to see that everything is over and the baby is in my lap."*

#### Depression

Feeling depressed was another experience expressed by 90% of the participants. The need to use a third party for fertility, recurring medical expenses and multiple failures, blame of others, lack of comprehensive support from the husband, and other destructive feelings and behaviors had led to depression in women. Participant 7 said in a hateful voice: *"I'm depressed now. I don't enjoy anything. Nothing makes me happy. This depression of mine has affected my husband too. He does not want to live with a depressed person in an unhappy and gloomy house. We are far apart".*

#### Spiritual discouragement

A kind of despair and spiritual discouragement was expressed by 50% of the participants. Women who had a strong relationship with spirituality before the offer to use donated oocytes had experienced this spiritual discouragement caused by problems such as multiple treatment failures and failure to be pregnant using autologous oocyte and, the need to use a third party in fertility. For example, participant 20 said in tears: *"I was always waiting for a miracle to happen and I thought that finally my prayers would be answered and we would have a child; but now I'm at the end of my rope, and think that my prayers are no longer effective and useful. I can't even have a cell of my own to be able to get pregnant."*

#### Social Stigmatization

The category of social stigmatization consisted of the three subcategories of concerns about disclosure, judgment of others, and conflict with religious teachings.

#### Disclosure

Concerns about disclosure and the consequent attempt to conceal the process of the treatment were among the predominant (95%) approaches of the study's participants. In addition to enduring the emotional burden of childlessness, these women were forced to hide the use of donated oocytes as a treatment method from others as they were concern about some social stigma. Participant 17 expressed her desire for keeping secret her use of donated oocyte as follows:

We didn't want anyone to know about it. We were severely under pressure. We couldn't consult anybody. We never said the details to those around us. We just said that we are under treatment. If we let others know about it, we should tolerate their scoffs and scolding too, and this stigma would stay with us forever.

#### Judgment of others

Judgment of others was also expressed by 55% of the participants. Concerns about the judgment of others for using donated oocyte, fear of the negative reactions of others to this technique, and stigmatizing them as the cause of infertility presented challenges to these women with regard to talking about this issue with others, especially their family. Moreover, concerns about the apparent dissimilarity of the resulting offspring to the parents and, consequently, the negative judgments of others together with tolerating some stigmas were among the important concerns of infertile oocyte recipient women. Most of the participants talked about the overwhelming pressure of others' judgments on their treatment and using donated oocytes. They were always judged by annoying questions such as why don't you have children? Why don't you treat? Or why don't you use this method of treatment? Participant 7 said: *"The judgment of others with regard to donated oocyte was very cruel. They used to say you wouldn't understand the feelings of a mother till you become mother or give birth to a child. But, what about now?! If they come to know that I have used donated oocyte and the baby's gene is not mine, they probably will say that you cannot understand the feelings of a mother until you have your own child…".*

With regard to the judgment of others, participant 13 said that *"My husband's family often stigmatized me, saying that I am infertile; I couldn't have children; my oocytes are weak and, thus, I have to use the oocytes of another woman."*

#### Conflict with religious teachings

Conflict with religious teachings was another challenge experienced and expressed by 35% of the participants. Before making sure that the use of donated oocytes did not contradict the religious rules of Islam, a number of participants considered it to be unethical and against the Sharia. In this regard, participant 7 said: *"We are a religious community. It is important that to whom this child is mahram. The resulting offspring is mahram to the paternal family, but what about maternal family? We are so religious and it is an important issue for us; that if my child is a boy, is he mahram to my mother and sister or not? This issue, if resolved for me, is still unresolved for our relatives."*

#### Negative coping mechanisms

Denial and aggression were two other subcategories from which the main category of negative coping mechanisms was extracted.

#### Denial

Behaviors such as visiting different physicians, performing multiple tests in different centers, interrupting treatment process or requesting other treatment methods with the hope that previous diagnoses have been wrong and to obtain a positive result were reflective of the psychological reaction of denial in 60% of the participants. Participant 1 stated in this regard*: "When the doctor told us that my oocytes were weak and that we should use donated oocytes, we were very disappointed … My husband had come to terms with this issue to some extent, but I still did not believe it … The doctor must be wrong as we have not had similar problems in our family; my mom get pregnant at the age of 48…".*

#### Aggression

Aggression was another behavior in 40% of the participants, which was caused by the insistence of others for having children or their disagreement with using donated oocyte. In such a condition, the participants had lost the control of their behavior and emotions and behaved aggressively. Participant 9 said: *"Even thinking about being pregnant by using someone else's oocyte made me quite nervous and aggressive; I reacted to my husband's words aggressively. I even threatened him once that if he didn't agree to use donated oocyte, I would get a divorce."*

## Discussion

The present study aimed to gain an understanding of the experiences of oocyte recipient women. The results of the study showed that social taboos resulting from the significance of fertility led to some psychological responses in oocyte recipients. One of the experiences expressed by these women was the destruction of self-esteem caused by the need to use non-autologous oocyte requiring the entry of a third party (donor) in the infertility treatment process. This experience was emanated from a woman's realization that she is unable to play her gender role and become a mother. Although the reproductive role of women is not limited to gamete sharing and the process of pregnancy and childbirth has an important function in reproduction, the lack of participation in the creation of offspring is of particular significance in different cultures [26].

The results showed that cultural teachings with regard to the definition of motherhood have been effective in destroying the participants' self-esteem. Concern about the judgments of others was indicative of the social pressures caused by the negative attitude of society, which could reduce the self-esteem of oocyte recipients, leading to their anxiety, stress, and depression. Increased anxiety and depression have been previously reported in infertile women [27].

However, according to studies, these psychological symptoms are reduced in the users of assisted reproductive techniques who get successfully pregnant using autologous oocytes [28]. Based on the results of the present study, oocyte recipients face some challenges which can continue even after a successful treatment. According to the results, concern about the judgment of others had caused them to worry about disclosure of the donation information as well as the genetic origin of the offspring. Not only did this issue cause anxiety in these women [29, 30], it was also at odds with religious beliefs.

Issues such as the resulting offspring's relationship with other family members and what in Islam is called mahram are other important issues whose observance requires providing others with information about the genetic origin of the offspring. Mahram is a significant issue in Islam. A mahram in Islam is a member of one's family with whom marriage is considered haram (illegal) and from whom the concealment of body with hijab is not obligatory. According to this religious belief, men and women who are not mahram with each other need to observe some restrictions.

This Islamic rule requires a woman to cover her body against a non-mahram man. Being mahram is determined based on the genetic link between family members. Therefore, in Muslim families, in order to comply with this principle, the use of donated oocytes for the treatment of infertility should be disclosed, so that those who are not genetically connected with the offspring and are not considered mahrams can observe the religious rules.

The conflict between the issue of being mahram and the preference of keeping secret the use of donated oocyte is one of the reasons for some ongoing psychological burdens throughout the life and for years after the birth of the offspring [31].

Depression and the resulting problems together with the loss of hope for having a child with a genetic similarity, that is symbolically valuable to women, led to a kind of spiritual discouragement in these women. As shown in this study, after learning about their inability to conceive with an autologous oocyte, this special group of recipients of ART had various feeling towards it. As such, they looked doubtfully and disappointedly at spirituality-related issues.

For instance, according to some women their infertility was due to their past sins and, thus, they believed disappointedly that their prayers would not be answered. However, as shown in a qualitative research, relying on a superior being (God) is one of the coping strategies to which Iranian women resort with regard to their infertility to gain power and manage this undesirable situation [32]. Thus, spiritual discouragement of these women suggests that they are more prone to psychological problems than other infertile women. In order to reduce these negative feelings and achieve mental health in all aspects, paying attention to spirituality and other related issues seems to be necessary.

Denial and aggression of the participants were among other findings of the study which have been reported in other studies on infertile couples as well [33, 34]. As revealed in study, after passing the stage of infertility denial through the diagnosis that they are unable to produce gametes and need to use donated oocyte, these women employ the mechanism of denial to cope with the caused stresses.

Moreover, the influx of other negative emotions such as the fear of a third party's entering their life and the subsequent feeling of disintegration of marital life, lack of control over their affairs and other problems which are different from those of infertile women treated with other ARTs made the women of this study use aggression as a negative reaction to their emotions.

It was also shown that oocyte recipients had some negative experiences which might affect their mental health. Accordingly, social support is one of the pivotal needs of these women because, while trying not to disclose the use of donated oocyte, they are deprived of any social support and psychological counseling. Accordingly, considering the socio-cultural context of their society, infertility service providers need to provide these women with appropriate counseling programs to reduce of the feeling of inability to control the affairs in them.

One of the limitations of this study was the non-inclusion of the women who needed to use donated oocyte but did not enter the process of the treatment as they denied their need. Additionally, this research was conducted in a society with specific Islamic beliefs and, thus, the results cannot be generalized to other societies.

## Conclusion

Investigating the experiences of oocyte recipients, we showed that these women develop negative psychological symptoms such as, stress, anxiety and depression, spiritual coldness and loss of self-esteem. Concerns about disclosure of this treatment method, judgment of others, and conflict between some cases of oocyte donation and religious beliefs were other negative feelings expressed by the participants of the study. Finally, it was found that these women may use maladaptive coping techniques, such as denial and aggression, following their treatment.

## Data Availability

Data and material are available on request from the corresponding author.
